# Anti-Inflammatory Activities of Licorice Extract and Its Active Compounds, Glycyrrhizic Acid, Liquiritin and Liquiritigenin, in BV2 Cells and Mice Liver

**DOI:** 10.3390/molecules200713041

**Published:** 2015-07-20

**Authors:** Ji-Yeon Yu, Jae Yeo Ha, Kyung-Mi Kim, Young-Suk Jung, Jae-Chul Jung, Seikwan Oh

**Affiliations:** 1 Department of Molecular Medicine and Tissue Injury Defense Research Center, School of Medicine, Ewha Womans University, Yangchon-ku, Seoul 158-710, Korea; E-Mails: melonlemon05@naver.com (J.-Y.Y.); mmac82@naver.com (J.Y.H.); 2 Life Science Research Institute, Novarex Co., Ltd, Ochang, Cheongwon, Chungbuk 363-885, Korea; E-Mail: kkm3507@novarex.co.kr; 3 College of Pharmacy, Pusan National University, Pusan 609-735, Korea; E-Mail: youngjung@pusan.ac.kr

**Keywords:** licorice extract, oxidative liver damage, anti-inflammation, glycyrrhizic acid, liquiritin, hepatoprotective effect

## Abstract

This study provides the scientific basis for the anti-inflammatory effects of licorice extract in a *t*-BHP (*tert*-butyl hydrogen peroxide)-induced liver damage model and the effects of its ingredients, glycyrrhizic acid (GA), liquiritin (LQ) and liquiritigenin (LG), in a lipopolysaccharide (LPS)-stimulated microglial cell model. The GA, LQ and LG inhibited the LPS-stimulated elevation of pro-inflammatory mediators, such as inducible nitric oxide synthase (iNOS), cyclooxygenase-2 (COX-2), tumor necrosis factor (TNF)-alpha, interleukin (IL)-1beta and interleukin (IL)-6 in BV2 (mouse brain microglia) cells. Furthermore, licorice extract inhibited the expression levels of pro-inflammatory cytokines (TNF-α, IL-1β and IL-6) in the livers of *t*-BHP-treated mice models. This result suggested that mechanistic-based evidence substantiating the traditional claims of licorice extract and its three bioactive components can be applied for the treatment of inflammation-related disorders, such as oxidative liver damage and inflammation diseases.

## 1. Introduction

Oxidative stress is an imbalance between the production and scavenging of reactive oxygen and nitrogen species (ROS and RNS) and free radicals that can induce lipid peroxidation, DNA fragmentation and protein oxidation [1]. These damages result in the loss of membrane integrity, structural and functional changes in proteins and gene mutations [2]. Oxidative stress is critically involved in a variety of diseases. ROS are generated during the body’s metabolic reactions and can react with and damage some cellular molecules, such as lipids, proteins and DNA [3].

**Figure 1:**
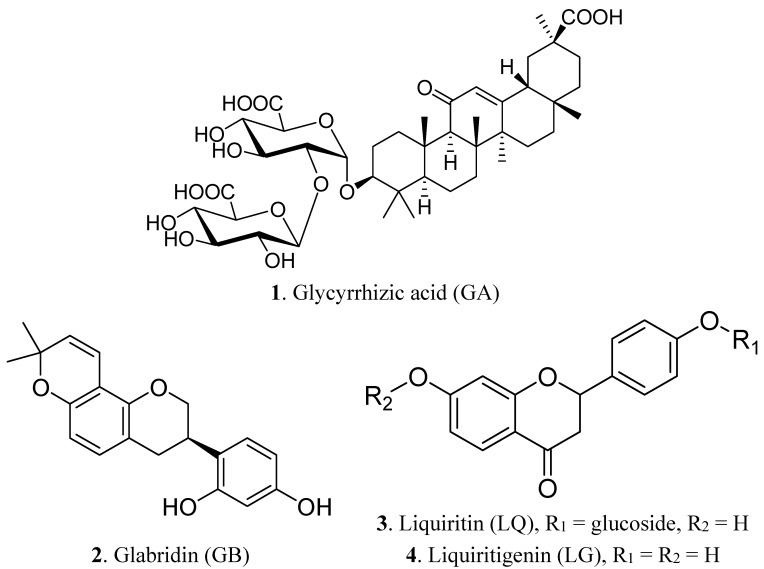
Structures of glycyrrhizic acid **1** (GA), glabridin **2** (GB), liquiritin **3** (LQ) and liquiritigenin **4** (LG).

Licorice (*Glycyrrhiza glabra*) is a traditional medicinal, sweet and soothing herb growing in several regions of the world. It is known that licorice has anti-inflammatory, anti-bacterial, antioxidative, anti-viral and expectorant properties [4,5,6] and is effective in the detoxification and protection of the liver [7]. The biologically-active components of licorice are well known as glycyrrhizic acid (GA, glycyrrhizin), liquiritin (LQ), glabridin (GB) and liquiritigenin (LG) (Figure 1). Recently, various studies have been preformed that analyzed and characterized the primary and secondary metabolites of licorice its active components [8,9]. Furthermore, the various biological effects of these compounds and the pharmacokinetics of the main component of licorice, GA, were reported [10].

Liver plays a pivotal role in the regulation of various physiological processes in the body, and one of the most important liver toxicity mechanisms might be a consequence of cell damage by ROS and RNS. Microglia, the characterized macrophages, are the major cellular target of inflammatory mediators in the central nervous system (CNS). Microglia can be stimulated during both neuroinflammatory and neurodegenerative disorders [11,12].

The aim of the present study was to evaluate the validity of the traditional therapeutic indications of licorice extract as a hepatoprotector and anti-inflammatory component. Therefore, we used *t*-BHP (*tert*-butyl hydrogen peroxide) to induce oxidative damage in mice and investigated the protective potential of licorice extract. LPS was applied and induced inflammation in microglia cell *in vitro* system. The protective effect of licorice extract on *t*-BHP-induced liver damage and inhibitory activities on cells may have important therapeutic meaning for the treatment of these inflammatory diseases.

## 2. Results and Discussion

### 2.1. Component Analysis of Licorice Extract

Licorice has been widely categorized as root twigs and root intercept, which are known for having representative biological compositions. We performed comparative experiments of the extraction for licorice sites using different solvents, such as water and 70% ethanol (in water), respectively, in laboratory conditions in order to provide a high concentration of index components, such as glycyrrhizic acid (GA), glabridin (GB), liquiritin (LQ) and liquiritigenin (LG). We focused on water and 70% ethanol (in water) for licorice extraction through confirmation of various organic solvent effects in terms of biological effectiveness and development as dietary supplements. 

On this test, the extracted licorice on the root twigs has a greater concentration of GA and LQ as the biologically-active components than that of root intercept. We found that the extraction of 70% ethanol for licorice root twigs showed our desired results of containing GA (1.68 ± 0.09, mg/g), GB (0.16 ± 0.02, mg/g), LQ (1.09 ± 0.04, mg/g) and LG (0.08 ± 0.01, mg/g) (Table 1). In addition, we found their contents of nutrients, such as carbohydrate (88.56%), protein (1.0%), fat (2.0%), ash (3.0%), sodium (0.996 mg/g) and moisture (4.44%). Interestingly, we found that GB is not detected in the water extract in either parts of licorice’s roots.

**Table 1:** Comparative active components of licorice extract.

Sample (Parts)	Solvent	GA (%)	GB (%)	LQ (%)	LG (%)
**1** (root twigs)	Water	0.75 ± 0.02	ND ^1^	0.26 ± 0.02	0.07 ± 0.01
**2** (root twigs)	70% EtOH	1.68 ± 0.09	0.16 ± 0.02	1.09 ± 0.04	0.08 ± 0.01
**3** (root intercept)	Water	0.51 ± 0.01	ND ^1^	0.15 ± 0.01	0.04 ± 0.001
**4** (root intercept)	70% EtOH	1.09 ± 0.01	0.08 ± 0.01	0.50 ± 0.01	0.01 ± 0.002

^1^ ND: not detected. The results are the means ± SD of three separate experiments.

### 2.2. HPLC-MS Analysis of Licorice Extracts

**Figure 2:**
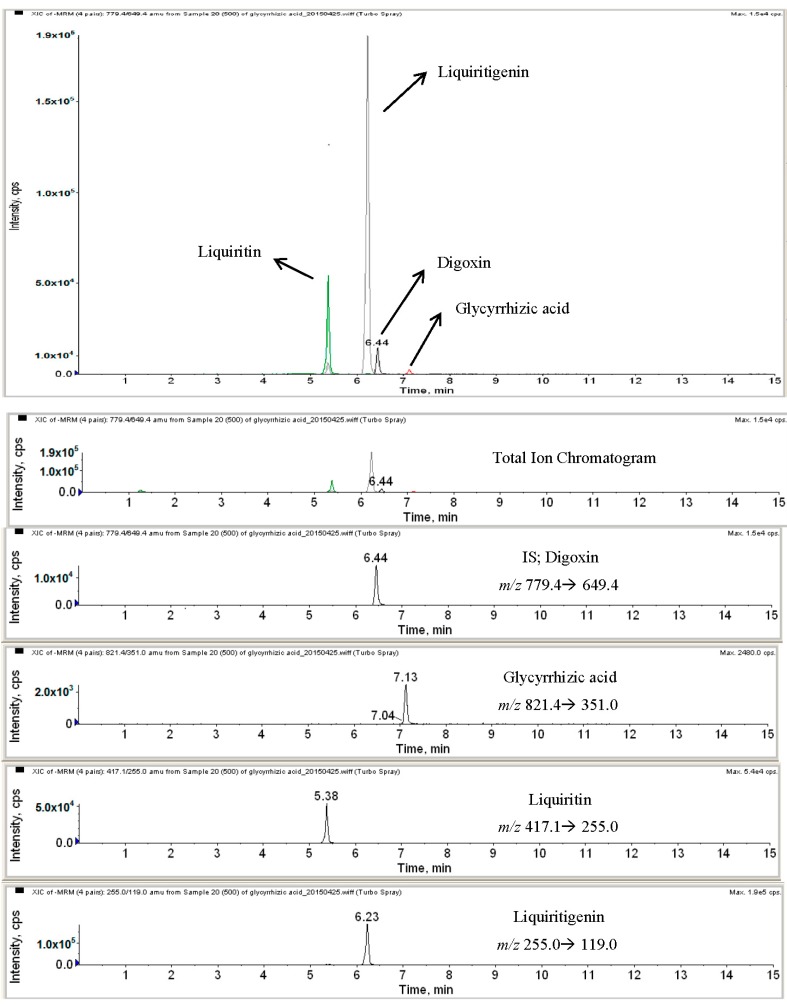
HPLC-MS analysis of licorice extracts.

We confirmed the active components of licorice extracts according to HPLC-MS analysis, as shown in Figure 2. GA, LQ and LG were detected at 7.13, 5.38 and 6.23 for the HPLC retention time, as well as they were also confirmed by MS at 821.4, 417.1 and 255.0 *m*/*z*; respectively.

### 2.3. Antioxidative Activity of Licorice Ingredients

**Figure 3:**
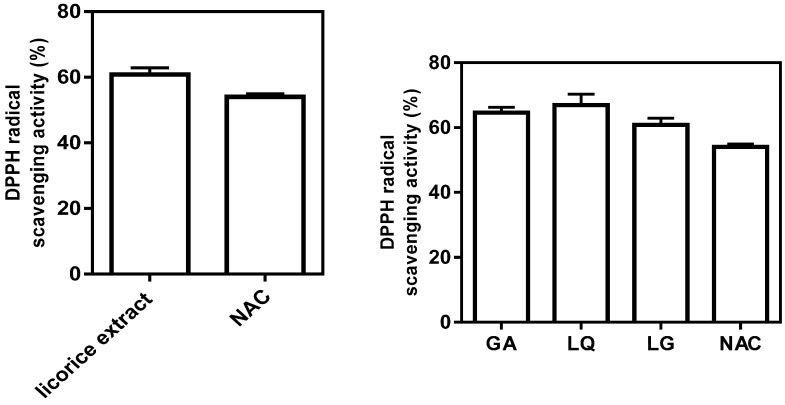
Free-radical scavenging activity of licorice extracts and three ingredients were measured by using the DPPH assay. The direct scavenging activity of licorice extract and its ingredients (GA, LQ and LG) on DPPH radicals was expressed as the % of control at 10 mg/mL of licorice root extract and 100 μM of GA, LQ and LG. NAC (*N*-acetyl cysteine) 10 μM was used as positive control. The results are the means ± SD of three separate experiments.

Numerous studies have shown that the beneficial effects of medicinal plants are closely associated with the presence of phenolic compounds [13]. It is known that compounds with antioxidant properties may exert anti-inflammatory effects. We therefore measured the antioxidative effect of licorice extract and its three active ingredients using a cell-free system. The licorice extract and three compounds revealed the potential to scavenge DPPH free radicals. These data demonstrated that licorice extract and three compounds, GA, LQ and LG, have antioxidative effects (Figure 3).

### 2.4. GA, LQ and LG Inhibited NO Production and Decreased the Expression Levels of iNOS and COX-2

**Figure 4:**
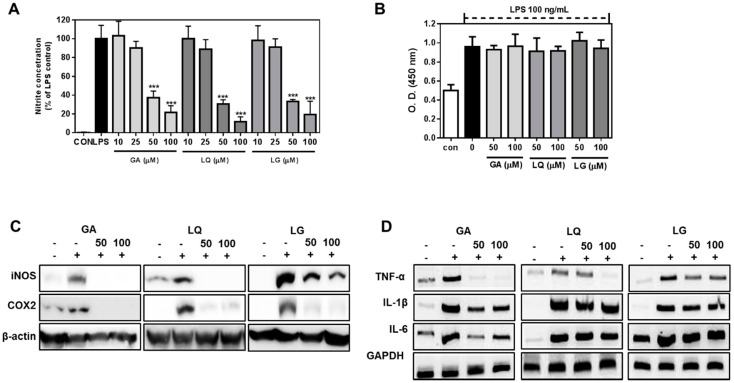
GA, LQ and LG suppressed the NO production and pro-inflammatory gene expression in LPS-stimulated BV2 cells. (**A**) Cells were exposed to each compound (50 and 100 μM) for 24 h in the presence of 100 ng/mL LPS. Cell viability was measured by theCCK-8 assay kit; (**B**) Cells were exposed to each compound (10 to 100 μM) for 18 h in the presence or absence of 100 ng/mL LPS. NO production was measured as nitrite concentration in the culture media; (**C**) Cells were stimulated with 100 ng/mL LPS in the presence of 50 and 100 μM GA, LQ and LG. After 18 h, the levels of iNOS and COX-2 were measured by Western blotting; (**D**) Cells were exposed to 50 and 100 μM GA, LQ and LG plus 100 ng/mL LPS for 18 h; the level of pro-inflammatory cytokine was determined by quantitative PCR. The results are the means ± SD of three separate experiments. *** *p* < 0.001 compared to the LPS-treated group.

GA, LQ and LG inhibited the LPS-stimulated NO production in a dose-dependent manner (Figure 4A). LPS-induced NO production was strongly suppressed with 50 μM and 100 μM of three compounds without cellular toxicity (Figure 4B). This suggests that GA, LQ and LG could potentially suppress microglial cells, which were activated by a variety of endotoxins. Furthermore, GA, LQ and LG inhibited the iNOS and COX-2 protein expressions in LPS-stimulated cells (Figure 4B). GA, LQ and LG blocked the NO production by preventing iNOS expression in LPS-exposed microglial cells. NO production affects the pro-inflammatory cytokine secretion and expression in cellular and serum level [14]. Therefore, we determined the expression levels of pro-inflammatory genes, TNF-α, IL-1β and IL-6. The genes were highly expressed in the LPS-stimulated BV2 cells, but the expression of TNF-α, IL-1β and IL-6 was significantly decreased by treatment with GA and LQ. However, the expression levels of IL-1β and IL-6 were inhibited by LG, but the TNF-alpha expression was not in the LPS-stimulated BV2 cells. These result showed that bioactive compounds of licorice extract have anti-inflammatory activities through the inhibition of pro-inflammatory cytokine expression.

### 2.5. Hepatoprotective Effect of Licorice Extract on the t-BHP-Induced Damage in Mice Liver

**Figure 5:**
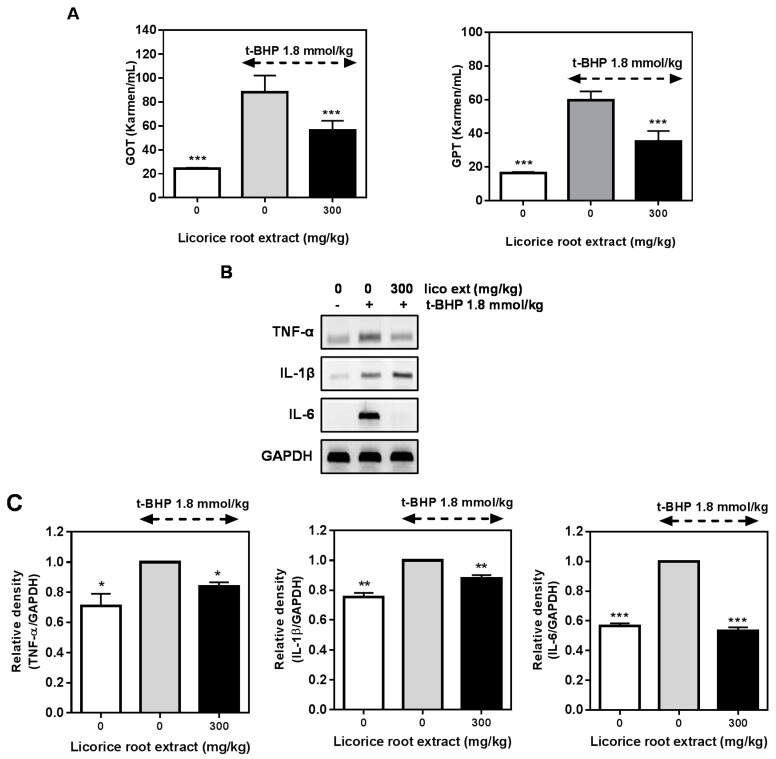
Hepatoprotective effect of licorice extract on the *tert*-butyl hydrogen peroxide(*t*-BHP)-induced liver damage in mice. (**A**) Plasma levels of the hepatic enzymes ALT and AST were determined using appropriate kits after *t*-BHP treatment for 24 h; (**B**) Animals were pre-treated with licorice extract for three days. The mice were administered *t*-BHP intraperitoneally on Day 4. After 24 h, the animals were euthanized by heart perfusion with saline, and the liver tissue was obtained. The levels of pro-inflammatory cytokines on liver tissue were determined by quantitative PCR; (**C**) Each pro-inflammatory cytokine expression was analyzed by using the relative density of GAPDH. The results are the means ± SD of three separate experiments. * *p* < 0.05, ** *p* < 0.01, *** *p* < 0.001 compared to the *t*-BHP only-treated group.

Biochemical measurement of liver function revealed that *t*-BHP treatment induced a significant increase in ALT (alanine aminotransferase) and AST (aspartate aminotransferase) levels in plasma, reaching 90 and 60 Karmen/mL values, respectively (Figure 5A). These values were reasonable evidence to explain the liver damage. The treatment of mice with *t*-BHP significantly increased the serum concentrations of ALT and AST, while these levels were attenuated by co-treatment with licorice extract in mice. The levels of pro-inflammatory cytokine TNF-α, IL-1β and IL-6 mRNA expressions were increased by the injection of *t*-BHP (1.8 mmol/kg) in mice. Those cytokine expressions were suppressed by the treatment of licorice roots extract (Figure 5B,C). These results show that licorice extract has anti-inflammatory and hepatoprotective effects against the *t*-BHP-induced oxidative damage in mice liver.

Recently, there has been a global trend toward the use of natural phytochemicals present in natural resources, such as fruits, vegetables, oilseeds and herbs, as antioxidants and functional foods [15,16]. Natural antioxidants can be used in the food industry, and there is evidence that these substances may exert their antioxidant effects within the human body [17].

The antioxidant and free radical scavenging activities of many substances have been assessed and have been found to exert anti-hepatotoxic effects related to strong antioxidant activity [18,19]. Recent studies have reported that licorice has various bioactive effects, including anti-inflammatory and antioxidative activity [20,21]. Licorice extracts have well-known superior efficacy in many metabolic diseases so far. In particular, the group of Jeong reported that 18-β-glycyrrhetinic acid and the aglycone of glycyrrhizin showed good liver protective effects for carbon tetrachloride-induced hepatotoxicity [22].

The indirect evidence of the reactive oxygen species (ROS) scavenging activity of licorice extract and three bioactive compounds was further confirmed using a direct approach with DPPH radicals. In this assay, the licorice extract and its ingredients (GA, LQ and LG, 100 μM) exhibited strong DPPH radical scavenging activity, and the activity was similar to that of 10 μM of *N*-acetyl cysteine (NAC), suggesting that licorice extract and its active compounds could be applied as natural antioxidants. *t*-BHP can be metabolized by cytochrome P450 or free iron ions, leading to the formation of free radical intermediates that initiate lipid peroxidation. The free radicals affect cell integrity and form covalent bonds with cellular molecules, resulting in cell death [23]. These phenomena are similar to the effects of sustained oxidative stress occurring in the cells and/or tissue [24,25].

Based on these studies, we investigated the potential protective effects of licorice extract in an experimental liver damage animal model that was induced by *t*-BHP treatment. To determine the anti-inflammatory effect of licorice ingredients, three single molecules, such as GA, LQ and LG, were assessed on the BV2 cells after induction of inflammation with LPS.

Elevation of ALT and AST levels in response to *t*-BHP has been attributed to hepatic structural damage, because these enzymes are normally localized to the cytoplasm and are released into circulation after cellular damage has occurred. Pretreatment of mice with licorice extract significantly suppressed the *t*-BHP-induced hepatotoxicity, as indicated by significant decreases of serum ALT and AST serum levels compared to the *t*-BHP-treated group (Figure 4A). It has been known that *t*-BHP induces oxidative stress and inflammation [3]; we examined whether *t*-BHP-induced pro-inflammatory cytokine expressions were decreased by licorice extract or not. The expressions of TNF-α and IL-6, but not IL-1β, were inhibited by the licorice root extract pre-treatment. These result explained that licorice extract has antioxidative and inflammatory effects in *t*-BHP-induced liver damage model.

Macrophages play key roles in common mechanisms of inflammation, namely over-production of NO by iNOS expression and secretion of cytokines, such as TNF-α, IL-1β and IL-6 [26]. Persistent NO production by macrophages could lead to carcinogenicity, cytotoxicity and autoimmune diseases [27,28]. COX-2 is involved in inflammatory responses, mediating the production of prostaglandins [29]. Therefore, a selective inhibition of iNOS and COX-2 is regarded as the most efficient approach to alleviating inflammation. Thus, the selective inhibition of macrophage activation is considered as an efficient approach to alleviating a variety of disorders triggered by inflammatory mediators. Herein, we demonstrate that the active components of licorice extract, GA, LQ and LG, strongly inhibited NO production in LPS-activated mice microglial cells. These compounds also almost strongly suppressed the expressions of TNF-α, IL-6 and IL-1β in LPS-treated cells. In addition, three compounds attenuated the expression of COX-2 and iNOS in LPS-stimulated BV2 cells (Figure 3). These results suggest that licorice ingredients (GA, LQ and LG) inhibit the generation of various inflammatory mediators produced by activated macrophages, and licorice root extract could be a good source to suppress inflammation in liver.

## 3. Experimental Section

### 3.1. BV2 Microglial Cell Culture

The murine BV2 cell line (a generous gift from W. Kim, KRIBB (Korea Research Institute of Bioecience & Biotechnology), Daejeon, Korea), which becomes immortal after infection with a *v-raf/v-myc* recombinant retrovirus, exhibits phenotypic and functional properties of reactive microglial cells. BV2 cells were maintained at 37 °C and 5% CO_2_ in Dulbecco’s Modified Eagle Medium (DMEM) supplemented with 10% heat-inactivated endotoxin-free FBS, 2 mM glutamine, 100 µg/mL streptomycin and 100 µg/mL penicillin. BV2 cells were grown in 6-well plates at a concentration of 2 × 10^5^ cells/well, followed by proper treatment.

### 3.2. Chemicals and Reagents

DPPH (2,2-diphenyl-1-pycrylhydrazyl), Griess reagents, *tert*-butyl hydrogen peroxide (*t*-BHP), glycyrrhizic acid, liquiritin and liquiritigenin were obtained from Sigma Co. (St. Louis, MO, USA). Dulbecco’s Modified Eagle’s Medium (DMEM), TRIzol, antibiotics and fetal bovine serum were obtained from Life Science Technology (Grand Island, NY, USA).

### 3.3. The Original Material

The raw material of licorice was cultivated in Jecheon-gun, Chungbuk province, Korea. The stocked raw licorice was qualified through separation and removal of foreign materials by the Department of Quality Control at Tecos (Jecheon, Korea). This was supplied from Jecheon licorice Co., Ltd. (Jecheon, Korea), in 2013.

### 3.4. Extraction and Isolation 

The dried licorice root (1 kg) was extracted with 70% ethanol (in water) (10 kg) at refluxed for 3 h, and then, the mixture was cooled to 30–35 °C. The mixture was filtered by a 75-µm cartridge and then treated with a centrifuge (15,000 rpm). The residue was vacuum concentrated at 55–58 °C to reach 60 brix materials. The mixture was homogenized by stirring and combined with dextrin. The combined mixture was spray died (liquid temperature: 75–80 °C; blow temperature: 180 °C; Atomizer 18,000 rpm) and then finally sterilized at 95 °C for 30 min to yield isolated licorice ingredients (240 g).

### 3.5. Analysis of HPLC-MS Instrument and Conditions

HPLC analysis was performed on a NANOSPACE SI-2 HPLC with HTS autosampler Z (Shiseido, Tokyo, Japan). The samples were separated on a reverse phase LUNA C18 column (2.0 × 150 mm, 5 μm) (Phenomenex, St. Louis, MO, USA). The sample was resolved using a linear gradient from 10% mobile Phase A (water containing 1% acetic acid) at a flow rate of 0.3 mL/min for 1 min, to 90% mobile Phase B (MeOH containing 1% acetic acid) over 6.5 min, followed by 90% mobile Phase B for 1.5 min. The column was then equilibrated for 6.5 min with 10% mobile Phase B. The HPLC column effluent was introduced onto an API 3200 Triple quadrupole mass spectrometer (ABCIEX, Toronto, ON, Canada) and analyzed using electrospray ionization in negative mode. Following the optimization of the setting parameters, the mass spectrometer was operated in negative mode for glycyrrhizic acid, liquiritin, liquiritigenin and digoxin (IS). The MS conditions were as follows: source temperature, 400 °C; curtain gas pressure, 20 psi; nebulizing gas (GS1) pressure, 40 psi; heating gas (GS2) pressure, 50 psi. Quantification was obtained using multiple reaction monitoring (MRM) mode in two MS/MS scan segments, monitoring the transitions of *m*/*z* 841.4→351.0, 417.1→255.0, 255.0→119.0 and 779.4→649.4, with the optimized collision energies and fragment voltages of −30 V, −28 V, −30 V and −44 V for glycyrrhizic acid, liquiritin, liquiritigenin and digoxin (IS). Data were acquired using Analyst 1.4.2 software (ABCIEX, Toronto, ON, Canada).

### 3.6. Animals and Treatment

Housing and experimental treatment of the animals were in accordance with the Institutional Animal Care and Use Committee of School of Medicine, Ewha Womans University. Adult male ICR mice (DaehanBioLink, Umseong, Korea), weighing 30–35 g, were used in the experiments. The mice were divided into six groups (6 mice/group) and were housed in polyethylene cages, lined with wood shavings, with wire mesh at the top, at an ambient temperature of 20 ± 2 °C, humidity between 40% and 60%, under a 12 h/12 h light/dark cycle. Food and drink were available *ad libitum*, except on the *t*-BHP-injection day, when all groups of animals were deprived of food. Body weight was monitored daily.

To study the protective effect against *t*-BHP-induced hepatotoxicity, licorice extracts was dissolved in saline (300 mg/kg body weight) and orally administrated to mice for three consecutive days. The extract concentrations were obtained according to the mean of body weights in each group. On Day 4, a saline solution (0.9% NaCl) of *t*-BHP (1.8 mmol/kg) was injected intraperitoneally into each animal, in a volume of 0.5 mL/100 g body weight. The control group was treated with saline solution. After 24 h, mice were euthanized by decapitation, and blood samples were collected for ALT and AST determination. The livers tissue were homogenized, and protein and mRNA were obtained.

### 3.7. 2,2-Diphenyl-1-picrylhydrazyl Radical Scavenging Activity

For the cell-free assay of antioxidative activity, the scavenging activity of licorice extracts, GA, LG and LQ, on 1,1-diphenyl-2-picrylhydrazyl (DPPH) free radicals was assessed according to the method reported by Gyamfi *et al*. [30]. Briefly, 100 mL of each compound (dissolved in 1 mg/mL) was mixed with 1 mL of 0.1 mM DPPH-ethanol solution and 450 mL of 50 mM Tris-HCl buffer (pH 7.4). After 30 min of incubation at room temperature, reduction of DPPH free radicals was measured by reading the absorbance at 517 nm.

### 3.8. Cell Viability Assay

Cell viability was measured by the CCK-8 assay (Ez-Cytox assay kit, Seoul, Korea). Initially, the cells were seeded into 96-well culture plates at 5 × 10^4^ cells/mL and cultured in DMEM containing 10% FBS at 37 °C for 24 h. When cells reached up to 70% confluence, the cells were treated with various concentrations (50 and 100 μM) of compounds. After 22 h of incubation, each well had 10 μL of CCK-8 reagent added and was incubated for 2 h. Then, they optical density was measured by spectrophotometry.

### 3.9. Nitrite Assay

NO production from activated microglial cells (BV2 cell lines) was determined by measuring the amount of nitrite, a relatively stable oxidation product of NO, as described previously [31]. Cells were incubated with or without LPS (1 µg/mL) in the presence or absence of various concentrations of compounds for 24 h. The nitrite accumulation in the supernatant was assessed by the Griess reaction. In brief, an aliquot of the conditioned medium was mixed with an equal volume of 1% sulfanilamide in distilled water and 0.1% *N*-1-naphthylethylenediamine dihydrochloride in 5% phosphoric acid. The absorbance was determined at 540 nm in an automated microplate reader.

### 3.10. Western Blot Analysis

Cell lysates were prepared in lysis buffer (50 mM Tris-HCl, pH 7.4, 1 mM EDTA, 150 mM NaCl, 1% Nonidet P-40, 0.25% Na-deoxycholate and 1 g/mL each of aprotinin, leupeptin and pepstatin), and the protein content was quantified according to the BCA protein quantification assay kit (Thermo Scientific, Waltham, MA, USA). Equal amounts of protein sample (30 μg/sample) were separated via 12% SDS-PAGE and blotted onto PVDF membranes. Blots were probed with primary antibodies and then incubated with horseradish peroxidase-conjugated anti-IgG in a blocking buffer for 1 h. The blots were developed with enhanced chemiluminescence (GE Healthcare, Buckinghamshire, UK). Polyclonal antibodies against iNOS, COX-2 and β-actin were obtained from Santa Cruz Biotechnology (Santa Cruz, CA, USA).

### 3.11. RNA Purification and Quantitative RT-PCR

Total RNA was extracted from cells using TRIzol reagent (Invitrogen, Carlsbad, CA, USA). The extracted RNA was quantified spectrophotometrically and reverse transcribed by AMV reverse transcriptase (Invitrogen, Carlsbad, CA, USA). Quantitative PCR was performed (PCR Master Mix, Bioneer, Daejeon, Korea) and analyzed with 7000 SDS v1.0 software. GAPDH was amplified to serve as an internal control to normalize the PCR efficiency, and the PCR primer sequence was designed and analyzed by primer express software (ThermoFisher Scientific, Waltham, MA, USA) (Table 2).

**Table 2:** Sequence and accession numbers for forward (F) and reverse (R) primers used in quantitative-PCR.

Gene	Accession No.	Sequences	Product Size (bp)
TNF-α	NM_012675.3	5′-CTGGCGTGTTCATCCGTTCT-3′ 5′-GACATTCCGGGATCCAGTGA-3′	283
IL-1β	NM_031512.2	5′-GAAGAGCCCGTCCTCTGTGA-3′ 5′-GTGGGTGTGCCGTCTTTCAT-3′	290
IL-6	NM_012589.2	5′-CCCACCAGGAACGAAAGTCA-3′ 5′-TCAGAATTGCCATTGCACAAC-3′	270
GAPDH	NM_017008.4	5′-ATGGTGAAGGTCGGTGTGAAC-3′ 5′-TGTAGTTGAGGTCAATGAAGG-3′	150

### 3.12. Statistical Analysis

Unless otherwise specified, all data are expressed as the mean ± standard deviation (SD) of three independent experiments. A one-way ANOVA using GraphPad Prism Version 6.0 software (La Jolla, CA, USA) was used for multiple comparisons. Values of * *p* < 0.05, ** *p* < 0.01, *** *p* < 0.001 were considered significant.

## 4. Conclusions

In conclusion, we found that bioactive compounds of licorice extract inhibited the production of NO and inflammatory cytokine in LPS-stimulated mouse microglial cells, and licorice extract showed a protective effect in the mice model of *t*-BHP-induced acute liver injury, which is in part related to their antioxidative activity. The protective potential of licorice extract and its three components (GA, LQ and LG) correlates directly with its antioxidant and anti-inflammatory properties, and licorice extract could be applied to treat inflammation-related diseases and oxidative liver damage.
